# Interaction between cognitive status, fear of falling, and balance in elderly persons

**DOI:** 10.6061/clinics/2020/e1612

**Published:** 2020-10-21

**Authors:** Graziela Morgana Silva Tavares, Bárbara Palma Pacheco, Maria Gabriela Valle Gottlieb, Daniela Virote Kassick M�ller, Gilmar Moraes Santos

**Affiliations:** I Universidade Federal do Pampa (Unipampa), Bage, RS, BR; II Universidade do Estado de Santa Catarina (UDESC), Florianopolis, SC, BR; III Pontificia Universidade Catolica do Rio Grande do Sul(PUC-RS), Porto Alegre, RS, BR

**Keywords:** Postural Balance, Accidental Falls, Elderly, Cognition

## Abstract

**OBJECTIVE:**

Evaluate the cognitive function and its relationship with balance, history of falls, and fear of falling in the elderly.

**METHODS:**

We evaluated 250 elderly persons aged at least 60 years, who answered a sociodemographic questionnaire about the occurrence of falls in the last year. The cognitive function, balance, and fear of falling were assessed using the Mini-Mental State Examination (MMSE), Berg Balance Scale (BBS), and Falls Efficacy Scale (FES-I) scores, respectively. Participants were allocated into two groups based on the Mini-Mental State Examination (MMSE) score, the Group with Possible Cognitive Decline (GPCD) and the group with no cognitive decline (GNCD). We performed Student's t-test and Pearson’s correlation for independent samples.

**RESULTS:**

The Group with Possible Cognitive Decline (GPCD) showed lower balance (*p*=0.003) and greater fear of falling (*p*=0.008) (BBS=50.98±4.68; FES-I=26.06±8.78) compared to the GNCD (BBS=52.53±3.047; FES-I=23.21±7.74).

**CONCLUSION:**

Elderly persons with cognitive decline have lower balance, greater fear of falling, and greater recurrence of falls.

## INTRODUCTION

Globally, there has been an increase in life expectancy with each population census, and this trend exists in Brazil as well. According to data recently published by the Brazilian Institute of Geography and Statistics-IBGE, 11.7% of the Brazilian population is aged at least 60 years, and this proportion is expected to double to 23.5% by 2039 (1).

Aging is a dynamic process that varies from one individual to another, and is characterized by a decline in physiological functions with decrease in the adaptation capacity in situation of functional overload (2).

The alterations that occur in the physical adaptation of the elderly include the loss of muscle strength and resistance, reduction in the range of motion, and gait modifications (3). Furthermore, nervous system changes may occur during the process of physiological aging. This predisposes the elderly to cognitive impairment, which is defined as the loss of the accuracy of skills such as language, memory, planning and execution of tasks, attention, and perception (2,4).

With the changes caused by aging, it becomes more difficult to main balance because the motor function involves an interaction of information from the visual, vestibular, and proprioceptive systems that are integrated in the central nervous system (CNS) (5). Thus, there is a decline in the postural control function, leading to greater motor variability, typically reflected in increased pressure center oscillation (6). Thus, these functional changes could predispose the elderly to balance disorders (7) and increase the tendency to fall.

Several studies (8-10) have been conducted in an attempt to understand the factors that are related to the increased propensity of falls and reduced balance, as well as their relationship with cognitive decline in the elderly. However, most of these studies were conducted on elderly residents in long-term care institutions, and studies conducted on community-based elderly residents remain scarce (11).

Given the above reasons, the present study aimed to evaluate the level of cognition and its relationship with balance, history of falls, and fear of falling in elderly individuals living in the city of Uruguaiana - RS, Brazil.

## MATERIAL AND METHODS

This was a cross-sectional, comparative, quantitative, and descriptive study. It was approved by the Human Ethics Committee of Pontifícia Universidade Católica do Rio Grande do Sul (PUCRS) (protocol numbers 312.127 and 930.945/15), and by the Ethics Committee of Universidade Federal do Pampa (Unipampa), (protocol number 2137.057), following the guidelines of Resolution 466/12 of the Brazilian Ministry of Health on Research involving humans.

After explaining the study protocol to the volunteers, they signed the free and informed consent form before participating in the study.

We included elderly individuals (60 years or above) who were able to stand and walk independently. We excluded individuals with a history of amputation and/or visual impairment or blindness.

### Variables and outcomes

In the present study, we investigated the following sociodemographic variables level of education, gender, age, income level, number of children, and marital status. These data were collected through a semi-structured questionnaire.

The following anthropometric parameters were investigated: weight, height, body mass index, waist circumference, hip circumference, and waist/hip ratio.

Participants were also asked about the occurrence and number of falls in the last 12 months.

For balance evaluation, we used the BBS, which measures the balance of individuals while practicing functional tasks. The scale has 14 tasks with the scores ranging from 0 to 4 points for each task, and giving a total score value from 0 to 56 points. Individuals who attain a score of 56 have a lower risk of falls compared to those who score below 56. The decrease in BBS score is associated with an increased risk of falls. In the ranges of 56 to 54 and 54 to 46, a one-point change in the BBS score is associated with a 3-4% and 6-8% increase in risk of falls, respectively (12,13).

The FES-I was to assess the fear of falling in the elderly during the performance of various daily activities. The scale is composed of 16 questions, each having a score from 1 to 4. Thus, the total score ranges from 16 to 64 points, where 16 and 64 points correspond, respectively, to the absence of concern and extreme concern for falls while performing activities (14,15).

The MMSE, also known as the Mini-Mental, was used to screen for cognitive function decline, being one of the most used because it is quick to apply (about 10 min) and does not require specific materials. This instrument evaluates various domains such as: temporal and spatial orientation, immediate and recall memory, calculus, language-naming, repetition, comprehension, writing and drawing (16).

The MMSE score ranges from a minimum of 0 point, which indicates the highest degree of cognitive impairment, to a maximum of 30 points, which correspond to the best cognitive ability (12). The present study adopted the cutoff point (17) according to the level of education. If the score was below the expected level of education, the individual was classified as having a probable cognitive deficit. Probably cognitive impairment was defined as scores <19, <23, <24, and <28 points for individuals who were illiterate, with 1 to 3 years of schooling, with 4 to 7 years of schooling, and with more than 7 years of schooling, respectively (13).

### Data collection

Data were collected in Unipampa, Serviço Social do Comércio-SESC Uruguaiana, and in three basic health units in the municipality of Uruguaiana, from July 2013 to March 2018. Prior to the collections, the researchers were trained in order to standardize the application of the data collecting instruments.

First, personal and sociodemographic data were collected, followed by anthropometric data, then the history of falls and fractures. Thereafter, the MMSE, BBS, and the FES-I were applied.

### Statistical analysis

The Statistical Package for the Social Sciences software version 20.0 was used for statistical analysis. The volunteers were assigned into two groups according to the MMSE score. The elderly who scored below the expected level of education were allocated to a GPCD, whereas those who obtained the same or higher than the expected score were allocated to the GNCD. For independent samples, Student’s t-test was used to determine a significant difference between the BBS score, FES-I score and MMSE score in participants with and without cognitive decline. The chi-square test was used to compare categorical variables. Additionally, Pearson's correlation coefficient (r) was used to quantify the relationship between the three scores. For this, values of r=0.10 to 0.30, r=0.40 to 0.60, and r=0.70 to 1 were considered weak, moderate, and strong, respectively. In all statistical tests, a significance level of *p*<0.05 was adopted.

## RESULTS

The final study sample was composed of 250 participants, with an age range of 60-86 years and a mean age of 69.37±6.42 years. The GPCD and GNCD were composed of 134 participants (53.6%) with a mean age of 69.65±6.77 years, and 116 participants (46.4%) with a mean age of 69.60±6.01 years, respectively. Table 1 shows the demographic and socioeconomic data, number of falls in the last year, and the frequency of falls in the GPCD and GNCD.

There was no statistically significant difference between the groups, regarding anthropometric variables including body mass index (*p*=0.423), waist circumference (*p*=0.494), hip circumference (*p*=0.350), and relationship between waist/hip ratio (*p*=0.591).

We found a weak correlation between the BBS and MMSE, BBS and FES-I, and MMSE and FES-I scores, with Pearson’s correlation coefficients of -0.323, -0.219 and -0.137, respectively. These were different from zero at *p*<0.001. The coefficients of determination between balance and cognitive performance, and between balance and fear of falling were 0.138 and 0.086, respectively, indicating that 13.8% of the variability in the BBS score could be attributed to the MMSE score, whereas 8.6% of the BBS score variability could be attributed to the FES-I score (Table 2).

Table 3 shows the MMSE, BBS, and FES-I scores of the GPCD and GNCD. The difference in mean BBS and MMSE scores in the GNCD was greater than that in the GPCD. On the other hand, the FES-I score was lower in the GNCD compared to that in the GPCD. These differences were statistically significant.

Table 4 shows the domains specified in the MMSE in the overall sample, and in the GPCD and GNCD.

## DISCUSSION

In this exploratory research, we analyzed the associations of cognitive decline with balance changes and falls in community-dwelling elderly persons. This study is, therefore, relevant as the number of elderly persons is increasing worldwide, and cognitive and balance changes are highly prevalent, negatively affecting the quality of life of this segment of the population.

Our study showed that elderly persons in the GPCD had a greater risk of falls and fear of falling compared with those in the GNCD. The literature suggests that elderly persons with cognitive decline are five times more likely to experience falls than those without cognitive decline (18), since the main cognitive functions that contribute to postural control and balance maintenance are memory, attention, and orientation (19).

When comparing MMSE domains to cognitive decline, we noticed that the calculation of items presented the most alterations. The cognitive deficits usually observed during the aging process are the forgetfulness of recent events, difficulty in performing calculations, alterations in the ability to adopt non-risky attitudes in attentiveness, decreased concentration and reasoning, and slowness when performing fine motor activities (20).

In the present study, the average study time was 5.5±3.5 years, suggesting an association with the MMSE score, as the elderly persons may show more interest and motivation for some of the activities analyzed. In a systematic review (21) on the use of MMSE in the Brazilian population, the average education level was 5.372.17 years, which corroborates the findings of the present study. In Brazil, several studies (22,23) have suggested adjustments in the cutoff points that could consider levels of education that are more appropriate to the reality of our country. The Brazilian culture has a high sociodemographic diversity, a fact that cannot be overlooked, due to the high incidence of people with low education and illiteracy.

When we analyzed individuals who experienced falls in the last 12 months, there was no significant difference compared to the GPCD, suggesting that the motor and functional capacity of the individuals was not yet compromised, since most of them could perform their activities independently. However, there was a significant difference in the number of falls and their recurrences between the groups. There are approximately 400 different types of falls and risk factors (24). Implicit to a fall, we can find the combination between dysfunctions of systems and organs and the performance of external tasks (25).

It was found that elderly persons experience significant balance changes in both the GPCD and GNCD. Balance changes may be associated with dysfunctions of neural and osteoarticular systems that help maintain postural control (25). Thus, the sensory system plays a fundamental role in balance. In addition, the association of visual, proprioceptive, and vestibular information is fundamental in generating appropriate responses to maintain postural control; moreover, if there are alterations, there are balance deficits.

Regarding the fear of falling, the elderly in the GPCD showed greater fear compared to the elderly in the GNCD, and this fear was directly related to a decrease in the BBS score, leading to a decrease or loss of independence. Oliveira and Yoshitome (26) reported that the loss of independence is directly associated with injuries caused by the impact of a fall, or is an indirect consequence of the fear of suffering a new fall (post fall syndrome). This is a limitation linked to the loss of confidence in safely walking independently. In addition to the decline in their activities of daily living, the elderly may develop other changes such as depression, feelings of helplessness, and social isolation (27,28).

It should be noted that this study did not evaluate the functional capacity and quality of life of elderly individuals, factors that may be related to the effect of cognitive impairment on balance changes and their activities of daily living.

In addition, our study presented some limitations, such as the absence of sample size calculation. Moreover, some variables that were not investigated could possibly interfere with the interpretation of results, such as drug use and the fact that cognitive impairment compromises the responses to the scales used.

## CONCLUSIONS

The findings of the present study suggest that older people with possible cognitive decline have lower balance, greater fear of falling, and greater recurrence of falls. Thus, it is indispensable for health professionals to investigate cognitive deficits and to think about strategies aimed at preventing falls, as well as improving balance.

## AUTHOR CONTRIBUTIONS

Tavares GMS contributed in conceived and designed the analysis, collected and contributed the data and analysis tools, writting the paper, reviewed the final version. Pacheco BP contributed in conceived and designed the analysis, collected data, writing the paper. Müller DVK contributed in conceived and designed the analysis and collected data. Gottlieb MGV contributed in conceived and designed the analysis. Santos GM contributed the data and analysis tools, writting the paper, reviewed the final version and perfomed the analysis.

## Figures and Tables

**Table 1:** Demographic and socioeconomic characteristics of the GPCD and GNCD.

Variable	GPCD	GNCD	*p*
Gender			
Male/Female	32/102	35/81	0.316
Age (years)	69.65±6.77	69.05±6.01	0.465
Marital status			
Married	57	46	
Divorced	7	8	-
Single	15	19	
Widow(er)	55	43	
Education (years)	5.5±3.5	5.15±3.06	0.133
Income month			
1 minimum wage	84	64	
≥3 minimum wage	23	26	-
Without income	9	10	
Did not answer	18	13	
Falls			
No	81	74	0.601
Yes	52	41	
Frequency of falls in the last 12 months.			
1 to 2 falls	38	37	0.037*
3 ≥ falls	14	4	

Abbreviations: GPCD, Group with possible cognitive decline; GNCD, Group with no cognitive decline.

In the gender and falls variables, we applied the chi-square test for two nominal samples and in the age variable we used the Student’s t-test for independent sample.

* Significantly different frequency of falls between GPCD and GNCD.

**Table 2:** Pearson’s correlation between BBS, FES-I, and MMSE scores.

	BBS		FES-I		MMSE	
	r	*p*	r	*p*	R	*p*
BBS			-0.219	0.001	0.323	0.001
FES-I	-0.219	0.001			-0.137	0.005
MMSE	0.323	0.001	-0.137	0.005		

Abbreviations: BBS, Berg Balance Scale; MMSE, Mini-Mental State Examination; FES-I, Falls Efficacy Scale.

Pearson's correlation coefficient (r).

**Table 3:** Difference between GPCD and GNCD in the mean and SD of MMSE, BBS, and FES-I.

Variable/Groups (n)	GPCD (134)	GNCD (116)	*p*
MMSE	22.32±4.41	27.49±2.11	0.001
BBS	50.98±4.68	52.53±3.047	0.003
FES-I	26.06±8.78	23.21±7.74	0.008

Abbreviations: GPCD, Group with possible cognitive decline; GNCD, Group with no cognitive decline; BBS, Berg Balance Scale; MMSE, Mini-Mental State Examination; FES-I, Falls Efficacy Scale; SD, standard deviation.

Student’s t-test for independent sample.

**Table 4:** Descriptive analysis of MMSE in the total sample and in the GPCD and GNCD.

	Minimum	Maximum	Total sample	GPCD (134)	GNCD (116)	*p*
Temporal orientation	1	5	4.24±0.65	3.88±1.05	4.67±0.588	0.001*
Spacial orientation	1	5	4.65±0.74	4.45±0.922	4.89±0.345	0.001*
Record	0	3	2.90±0.37	2.82±0.488	2.98±0.131	0.001*
Calculation	0	5	3.22±1.95	2.28±1.990	4.33±1.190	0.001*
Evocation	0	3	2.07±0.90	1.82±0.916	2.37±0.787	0.001*
Language	3	9	7.69±1.27	7.22±1.336	8.24±0.942	0.001*

Abbreviations: GPCD, Group with possible cognitive decline; GNCD, Group with no cognitive decline.

Student t-test for independent sample.

## References

[1] Geografia IBGE, População ECd, Sociais I (2018). Síntese de indicadores sociais: uma análise das condições de vida da população brasileira, 2018: IBGE.

[2] Dziechciaż M, Filip R (2014). Biological psychological and social determinants of old age: Bio-psycho-social aspects of human aging. Ann Agric Environ Med.

[3] Souza LHR, Brandão JCS, Fernandes AKC, Cardoso BLC (2017). Queda em idosos e fatores de risco associados. Revista de Atenção è Saúde (antiga Rev Bras Ciên Saúde).

[4] Caixeta GC, Ferreira A (2009). Desempenho cognitivo e equilíbrio funcional em idosos. Rev Neurocienc.

[5] Sachetti A, Vidmar MF, da Silveira MM, Wibelinger LM (2012). Equilíbrio x Envelhecimento Humano: um desafio para a fisioterapia. Revista de Ciências Médicas e Biológicas.

[6] Rath R, Wade MG (2017). The Two Faces of Postural Control in Older Adults: Stability and Function. EBioMedicine.

[7] da Silveira MM, Pasqualotti A, Colussi EL, Wibelinger LM (2011). Envelhecimento humano e as alterações na postura corporal do idoso. Revista de Atenção è Saúde (antiga Rev Bras Ciên Saúde).

[8] Al-Momani M, Al-Momani F, Alghadir AH, Alharethy S, Gabr SA (2016). Factors related to gait and balance deficits in older adults. Clin Interv Aging.

[9] Zhang L, Zeng Y, Weng C, Yan J, Fang Y (2019). Epidemiological characteristics and factors influencing falls among elderly adults in long-term care facilities in Xiamen, China. Medicine (Baltimore).

[10] Baixinho CL, Dixe MdA, Madeira C, Alves S, Henriques MA (2019). Falls in institutionalized elderly with and without cognitive decline A study of some factors. Dement. neuropsychol.

[11] Machado JC, Ribeiro RdCL, Cotta RMM, Leal PFdG (2011). Declínio cognitivo de idosos e sua associação com fatores epidemiológicos em Viçosa, Minas Gerais. Rev bras geriatr gerontol.

[12] Alves NB, Scheicher ME (2011). Equilíbrio postural e risco para queda em idosos da cidade de Garça, SP. Rev. bras. geriatr. gerontol.

[13] Miyamoto ST, Lombardi Junior I, Berg KO, Ramos LR, Natour J (2004). Brazilian version of the Berg balance scale. Braz J Med Biol Res.

[14] Camargos FF, Dias RC, Dias JM, Freire MT (2010). Adaptação transcultural e avaliação das propriedades psicométricas da Falls Efficacy Scale-International em idosos brasileiros (FES-I-BRASIL). Revista Brasileira de Fisioterapia.

[15] Delbaere K, Smith ST, Lord SR (2011). Development and initial validation of the iconographical falls efficacy scale. J Gerontol A Biol Sci Med Sci.

[16] Hernandez SS, Coelho FG, Gobbi S, Stella F (2010). Efeitos de um programa de atividade física nas funções cognitivas, equilíbrio e risco de quedas em idosos com demência de Alzheimer. Rev. bras. fisioter.

[17] Adair N, Adams H, Adler J (2004). Current geriatric diagnosis and treatment.

[18] dos Santos ML, de Andrade MC (2005). Incidência de quedas relacionada aos fatores de riscos em idosos institucionalizados. Rev baiana saúde pública.

[19] da Cruz DT, da Cruz FM, Ribeiro AL, da Veiga CL, Leite IC (2015). Associação entre capacidade cognitiva e ocorrência de quedas em idosos. Cadernos Saúde Coletiva.

[20] Bertolucci PH, Minett TS, Prado FC, Ramos J, Valle JR (2007). Perda de memória e demência. Atualização terapêutica 23a ed. São Paulo: Artes Médicas.

[21] de Melo DM, Barbosa AJ (2015). O uso do Mini-Exame do Estado Mental em pesquisas com idosos no Brasil: uma revisão sistemática. *Ciênc.*. saúde coletiva [online].

[22] Scazufca M, Almeida OP, Vallada HP, Tasse WA, Menezes PR (2009). Limitations of the Mini-Mental State Examination for screening dementia in a community with low socioeconomic status: results from the Sao Paulo Ageing & Health Study. Eur Arch Psychiatry Clin Neurosci.

[23] Kochhann R, Cerveira MO, Godinho C, Camozzato A, Chaves MLF (2009). Evaluation of Mini-Mental State Examination scores according to different age and education strata, and sex, in a large Brazilian healthy sample. Dement Neuropsychol.

[24] Gschwind YJ, Wolf I, Bridenbaugh SA, Kressig RW (2011). Basis for a Swiss perspective on fall prevention in vulnerable older people. Swiss Med Wkly.

[25] Ricci NA, Gonçalves DF, Coimbra AM, Coimbra IB (2009). Sensory interaction on static balance: A comparison concerning the history of falls of community-‐dwelling elderly. Geriatrics & gerontology international.

[26] Ferreira DC, Yoshitome AY (2010). Prevalência e caraterísticas das quedas de idosos institucionalizados. Revista Brasileira de Enfermagem.

[27] Falsarella GR, Gasparotto LP, Coimbra AM (2014). Quedas: conceitos, frequências e aplicações è assistência ao idoso. Revisão da literatura. Revista Brasileira de Geriatria e Gerontologia.

[28] Rahman MS (2018). Prevalence and risk factors of fear of falling among elderly: A Review. Medical Journal of Clinical Trials & Case Studies.

